# 32 species validation of a new Illumina paired-end approach for the development of microsatellites

**DOI:** 10.1371/journal.pone.0081853

**Published:** 2013-11-28

**Authors:** Stacey L. Lance, Cara N. Love, Schyler O. Nunziata, Jason R. O’Bryhim, David E. Scott, R. Wesley Flynn, Kenneth L. Jones

**Affiliations:** 1 Savannah River Ecology Laboratory, University of Georgia, Aiken, South Carolina, United States of America; 2 Department of Biochemistry and Molecular Genetics, University of Colorado School of Medicine, Aurora, Colorado, United States of America; International Atomic Energy Agency, Austria

## Abstract

Development and optimization of novel species-specific microsatellites, or simple sequence repeats (SSRs) remains an important step for studies in ecology, evolution, and behavior. Numerous approaches exist for identifying new SSRs that vary widely in terms of both time and cost investments. A recent approach of using paired-end Illumina sequence data in conjunction with the bioinformatics pipeline, PAL_FINDER, has the potential to substantially reduce the cost and labor investment while also improving efficiency. However, it does not appear that the approach has been widely adopted, perhaps due to concerns over its broad applicability across taxa. Therefore, to validate the utility of the approach we developed SSRs for 32 species representing 30 families, 25 orders, 11 classes, and six phyla and optimized SSRs for 13 of the species. Overall the IPE method worked extremely well and we identified 1000s of SSRs for all species (mean = 128,485), with 17% of loci being potentially amplifiable loci, and 25% of these met our most stringent criteria designed to that avoid SSRs associated with repetitive elements. Approximately 61% of screened primers yielded strong amplification of a single locus.

## Introduction

Microsatellites, or simple sequence repeats (SSRs), are the genetic marker of choice for numerous applications in forensics, ecology, and evolution [1]. In particular their high variability and abundance across genomes make them ideal for studies of kinship, parentage, individual identification, population genetics, and linkage mapping (reviewed in [2]). In recent years, technological advances have brought other genetic markers into favor. For example, single nucleotide polymorphisms (SNPs) have gained favor for linkage studies [3], are increasingly being used in wildlife forensics [4], and with the development and improvement [5] of restriction-site associated DNA (RAD) tag sequencing approaches for SNP assays are likely to be increasingly used in population genetics studies (e.g., [6,7]). However, SSRs remain integral as is evidenced by examining a recent issue (vol 22 issue 4) of *Molecular Ecology* in which over 50% of the original articles relied on microsatellite analysis. In addition, new SSR loci are still being continually developed (e.g., 58 papers describing new SSR loci in *Conservation Genetics Resources* vol 4 no 4 December 2012).

Although SSR loci remain the genetic marker of choice, their development is still considered to be expensive and labor intensive. For many years, SSR development involved creating libraries enriched for repeat motifs, cloning the library, and using traditional Sanger sequencing to identify clones with inserts positive for SSRs. With the advent of next-generation sequencing technologies, methods for development and characterization of SSRs have improved dramatically. Most notably, researchers began using the Roche 454 sequencing platform to sequence SSR-enriched libraries [8]. Since then, our lab has used the enrichment and 454 sequencing methods in combination across a broad range of taxa including vertebrates [9-12], invertebrates [13-15], and plants [16,17]. While the two methods in tandem have worked well, the enrichment process is nonetheless time consuming, limits the search to selected motifs, can require high concentrations of DNA as starting material. In some species can result in inadvertent enrichment for transposable elements, which have similar motifs to SSRs [18]. It is possible to avoid inadvertent enrichment by employing shotgun sequencing on the 454 platform [19,20]; however, for species with large genomes or infrequent SSRs the cost can be prohibitive. Recently, a more cost effective and efficient method for SSR development using Illumina sequencing has been described [21]. Still, even with the technological advances of next-generation sequencing, the most common method for SSR detection still involves cloning and Sanger sequencing. In the SSR development papers in the issue of *Conservation Genetics Resources* mentioned above, the authors used Sanger sequencing in 52%, 454 sequencing (1/3 with enriched libraries) in 36%, and Illumina sequencing in only one article. 

In recent years, advances in Illumina sequencing have substantially increased the number of reads obtained. In addition, the cost of Illumina sequencing has decreased while the cost of 454 sequencing has remained stable. As a result, it is now cost efficient to use a shotgun sequencing approach with Illumina paired-end sequencing (IPE) 100 bp (HiSeq) or 150 bp (GAIIx) to identify SSRs [21]. Castoe et al. [21] demonstrate that for one species, the Burmese python, shotgun sequencing via IPE and 454 yielded similar results and that IPE reads worked well for two species of birds, even though birds have relatively low frequency of SSR loci [22]. Though Castoe et al. thoroughly describe the SSR data from the IPE reads, they did not validate the primers designed for the three species. The method described by Castoe et al. is highly promising; however, there are two major concerns for the IPE method. First, that the short reads may not allow for sufficient flanking sequence to design primers. Second, that when primers are designed there is no estimate of amplicon length because the two sequences from the paired-end read may not overlap, and thus numerous loci may be either too short or long for classical fragment analysis. Given the apparent hesitancy of researchers to switch to next-generation sequencing for SSR development, we sought to assess and validate the IPE method for a variety of taxa. Our objectives include 1) comparing two different IPE shotgun library preparation protocols (one that requires 1 µg of DNA and one that only requires 10 ng), 2) using the IPE approach across a broad range of taxa to assess the number of reads returned positive for SSRs, the number of positive reads suitable for primer design, and the types of SSRs identified, and 3) to validate that primers designed via IPE will produce quality SSR loci for genotyping purposes.

## Methods

### Library preparation and sequencing

Within a total of 32 species that comprise a wide taxonomic range (table 1), we used two different methods (16 species each) for creating Illumina paired-end shotgun libraries. The first entailed shearing 1 µg of genomic DNA using a Covaris S220, following the standard protocol of the Illumina TruSeq DNA Library Kit, and using the multiplex identifier adaptor indices. The second method followed the standard protocol of the Nextera™ DNA Sample Prep Kit from Epicentre^®^ that uses only 10 ng of genomic DNA and incorporates Illumina-compatible bar codes. With both methods we pooled 4 - 8 libraries and conducted Illumina sequencing on the HiSeq with 100 bp paired-end reads. We demultiplexed the raw data using Illumina's standard GERALD pipeline. Following demultiplexing, we quality controlled reads for each species to remove bad reads. We wrote a Python QC script (available at https://gist.github.com/jonesken/6226417) to: remove "B-tail" bases (strings of bases with qualities less than Q15 at the end of a read, denoted by the B quality score in Phred-64 data), remove trimmed reads less than 50 bp, and reduce the files to 5M QC-passed paired reads. The resulting reads were analyzed with the program PAL_FINDER_v0.02.03 [21] to extract those reads that contained perfect di-, tri-, tetra-, penta-, and hexanucleotide microsatellites and batch positive reads to a local installation of the program Primer3 (version 2.0.0) for primer design.

**Table 1:** Taxonomic information for the 32 species sequenced.

**Sample Number**	**Kingdom**		**Phylum**	**Class**	**Order**	**Family**	**Genus**	**Species**
**1**	Animalia	Arthropoda		Insecta	Coleoptera	Dytiscidae	*Stictotarsus*	*aequinoctialis*
2	Animalia	Arthropoda		Insecta	Hemiptera	Plataspidae	*Megacopta*	*Cribraria*
**3**	Animalia	Arthropoda		Insecta	Lepidoptera	Nymphalidae	*Junonia*	*coenia*
4	Animalia	Arthropoda		Insecta	Plecoptera	Capniidae	*Mesocapnia*	*arizonensis*
**5**	Animalia	Arthropoda		Malacostraca	Decapoda	Lithodidae	*Paralithodes*	*platypus*
**6**	Animalia	Arthropoda		Malacostraca	Decapoda	Ocypodidae	*Uca*	*mimax*
7	Animalia	Arthropoda		Malacostraca	Decapoda	Ocypodidae	*Uca*	*spinicarpa*
8	Animalia	Chordata		Actinopterygii	Cypriniformes	Cyprinidae	*Rhinichthys*	*osculus*
**9**	Animalia	Chordata		Actinopterygii	Salmoniformes	Salmonidae	*Prosopium*	*williamsoni*
**10**	Animalia	Chordata		Amphibia	Caudata	Ambystomatidae	*Ambystoma*	*talpoideum*
**11**	Animalia	Chordata		Amphibia	Caudata	Pletodontidae	*Eurycea*	*cirrigera*
**12**	Animalia	Chordata		Aves	Charadriiformes	Alcidae	*Alca*	*torda*
**13**	Animalia	Chordata		Aves	Charadriiformes	Alcidae	*Ptychoramphus*	*aleuticus*
14	Animalia	Chordata		Aves	Passeriformes	Troglodytidae	*Campylorhynchus*	*brunneicapillus*
**15**	Animalia	Chordata		Aves	Pelecaniformes	Pelecanidae	*Pelecanus*	*occidentalis*
16	Animalia	Chordata		Aves	Pelecaniformes	Sulidae	*Sula*	*bassanus*
**17**	Animalia	Chordata		Aves	Procellariiformes	Hydrobatidae	*Oceanodroma*	*castro*
**18**	Animalia	Chordata		Mammalia	Cetacea	Delphinidae	*Tursiops*	*truncatus*
19	Animalia	Chordata		Mammalia	Chiroptera	Phyllostomatidae	*Ectophyla*	*alba*
20	Animalia	Chordata		Mammalia	Didelphimorphia	Didelphidae	*Tlacuatzin*	*canescens*
21	Animalia	Chordata		Mammalia	Rodentia	Cricetidae	*Onychomys*	*leucogaster*
22	Animalia	Chordata		Reptilia	Squamata	Colubridae	*Lampropeltis*	*getula*
23	Animalia	Chordata		Reptilia	Squamata	Phrynosomatidae	*Sceloporus*	*grammicus*
24	Animalia	Chordata		Reptilia	Testudines	Geoemydidae	*Batagur*	*trivittata*
**25**	Animalia	Mollusca		Bivalvia	Unionoida	Unionidae	*Leptodea*	*Leptodon*
26	Plantae	Embryophyta		Equisetopsida	Asterales	Campanulaceae	*Canarina*	*n/a*
**27**	Plantae	Magnoliophyta		Magnoliopsida	Asterales	Asteraceae	*Solidago*	*gigantea*
**28**	Plantae	Magnoliophyta		Magnoliopsida	Caryophyllales	Cactaceae	*Echinocereus*	*n/a*
**29**	Plantae	Magnoliophyta		Magnoliopsida	Fabales	Fabaceae	*Lupinus*	*aridorum*
30	Plantae	Magnoliophyta		Magnoliopsida	Rosales	Rosaceae	*Bencomia*	*exstipulata*
31	Plantae	Magnoliophyta		Magnoliopsida	Scrophulariales	Scrophulariaceae	*Mimulus*	*ringens*
32	Plantae	Tracheophyta		Coniferopsida	Coniferales	Cupressaceae	*Juniperus*	*cedrus*

Sample number in bold indicates a Nextera library preparation method was used instead of the standard Illumina preparation.

### Primer Screening

For 12 of the 32 species, we tested forty-eight primer pairs for clean amplification and polymorphism across DNA obtained from eight individuals per species. We performed all PCR amplifications in a 12.5-μL volume (10 mM Tris pH 8.4, 50 mM KCl, 25.0 μg/ml BSA, 0.4 μM unlabeled primer, 0.04 μM tag-labeled primer, 0.36 μM universal dye-labeled primer, 3.0 mM MgCl_2_, 0.8 mM dNTPs, 0.5 units AmpliTaq Gold® Polymerase (Applied Biosystems), and 20 ng DNA template) using an Applied Biosystems GeneAmp 9700. For all loci, we used a touchdown thermal cycling program [23] encompassing a 10°C span of annealing temperatures ranging between 65-55°C. Touchdown cycling parameters consisted of an initial denaturation step of 5 min at 95°C followed by 20 cycles of 95°C for 30 s, 65°C (decreased 0.5°C per cycle) for 30 s, and 72 °C for 30 s; and 20 cycles of 95 °C for 30 s, 55°C for 30 s, and 72 °C for 30 s; and a final extension at 72°C for 5 m. We ran all PCR products on an ABI-3130xl sequencer and sized with Naurox size standard prepared as described in DeWoody et al. [24], except that unlabeled primers started with GTTT. We used GeneMapper version 3.7 (Applied Biosystems) to analyze alleles.

### Data Analysis

We performed all statistical tests using general linear models (GLM; SAS version 9.2, SAS 2009). We first tested the effect of library prep METHOD on the numbers of SSRs and PALs identified; with no difference in prep method detected, we removed METHOD from subsequent models. We tested for taxonomic effects on numbers of SSRs, PALs, and Premium PALs (see below) identified at the kingdom, phylum, and class levels. We calculated the proportions of repeat types (hexa-, penta-, tetra-, tri-, and dinucleotides) out of all SSRs, the proportions out of all PALs, and the proportion of Premium PALs to PALs—proportion data were arcsin-squareroot transformed prior to analyses for taxonomic effects. 

## Results and Discussion

To determine the overall efficiency of the method, we sequenced IPE libraries for 32 species across a wide taxonomic range (table 1; NCBI BioProject PRJNA209850). Overall the IPE method worked extremely well and we identified 1000s of SSRs for all species (mean = 128,485) with the fewest (2,541) found in a bird species and the highest (644,886) in a crab (table 2). Due to the relatively short read length of the IPE method as compared with Sanger sequencing or 454, the ability to identify suitable primer sites was a concern. However, enough suitable flanking sequence was available for primer design in 17% of the reads with SSRs yielding on average 19,072 potentially amplifiable loci (PALs, sensu [21]). Though 17% is not a large value, given the vast amount of data produced, the process results in ample PALs. The library preparation method did not impact either the number of microsatellites (F=0.07, p = 0.79) or the number of PALs identified (F= 0.05, p = 0.8176). Though the Nextera method is more expensive it allows for using the IPE method even when only 10 ng of DNA is available. The ability to use very small quantities of DNA can be very important for species in which only non-invasive samples can be used or DNA is difficult to extract. 

**Table 2:** The number of paired end reads out of 5 million that contain microsatellites, and within those the number that contain suitable sequence for primers and are considered potentially amplifiable loci (PALs).

**Sample Number**	**Genus**	**Number of sequences with microsatellites**	**Number of PALs**	**6mers**	**5mers**	**4mers**	**3mers**	**2mers**
**1**	*Stictotarsus*	50,735	2,576	1,333	3,413	6,072	3,946	35,971
2	*Megacopta*	86,717	13,953	28	122	2,408	6,674	77,485
**3**	*Junonia*	62,927	6,998	250	34,241	1,790	4,599	6,747
4	*Mesocapnia*	73,137	13,090	2,462	11,669	9,277	14,391	35,338
**5**	*Paralithodes*	430,868	54,838	350	194,790	20,956	51,573	163,199
**6**	*Uca*	644,886	144,502	70	13,010	42,400	199,907	389,499
7	*Uca*	545,301	94,805	114	13,360	40,449	88,638	402,740
8	*Rhinichthys*	238,812	30,099	2,796	1,560	106,375	9,013	119,069
**9**	*Prosopium*	286,604	26,109	140	257	1,943	3,374	20,395
**10**	*Ambystoma*	5,970	1,582	4	70	290	554	664
**11**	*Eurycea*	27,272	4,198	1,572	1,043	16,853	4,281	3,523
**12**	*Alca*	14,288	2,136	4,189	2,054	2,246	1,995	3,804
**13**	*Ptychoramphus*	17,166	3,093	26	274	608	1,444	741
14	*Campylorhynchus*	113,109	4,760	64,127	28,928	11,599	5,837	2,618
**15**	*Pelecanus*	12,421	2,554	2,450	3,459	1,344	3,032	2,135
16	*Sula*	82,003	3,913	4,275	69,353	1,684	4,531	2,160
**17**	*Oceanodroma*	2,541	418	592	390	217	646	696
**18**	*Tursiops*	34,387	6,999	2,150	301	4,110	2,411	25,415
19	*Ectophyla*	25,278	7,403	2,774	253	4,344	3,096	14,811
20	*Tlacuatzin*	94,285	12,811	3,865	2,821	36,927	13,016	37,656
21	*Onychomys*	132,502	33,500	86	316	4,433	3,817	24,848
22	*Lampropeltis*	244,857	26,215	302	4,144	8,975	5,967	6,827
23	*Sceloporus*	139,529	46,255	4,320	1,092	21,778	63,513	48,827
24	*Batagur*	22,319	6,370	19	71	486	1,146	4,648
**25**	*Leptodea*	105,238	8,601	4,015	606	44,611	13,035	42,971
26	*Canarina*	37,868	7,242	8	12	60	1,440	5,722
**27**	*Solidago*	31,634	7,607	75	405	405	4,555	2,167
**28**	*Echinocereus*	60,583	6,964	58	539	1,159	2,597	2,611
**29**	*Lupinus*	391,973	5,845	105	2,154	426	1,841	1,319
30	*Bencomia*	42,786	14,777	1,295	723	606	14,632	25,530
31	*Mimulus*	32,170	7,232	400	147	484	7,907	23,232
32	*Juniperus*	21,352	2,853	18	36	87	1,375	1,337

Also included are the number of those SSRs that contained hexanucleotide, pentanucleotide, tetranucleotide, trinucleotide, or dinucleotide repeats. Sample number in bold indicates a Nextera library preparation method was used instead of the standard Illumina preparation.

We further filtered the PALs to identify those for which both the forward and reverse primer sequences were found only one time throughout the 5 million reads. These loci are deemed the loci with the best potential for clean amplification and are considered the Premium PALs (hereafter referred to as pPALs). One problem with older enrichment methods is the inadvertent selection of SSRs associated with transposable elements [18]. It is well described that for some taxa SSRs often occur in repetitive elements. When primers are designed for these SSRs, they often amplify multiple loci and accurately scoring such loci can be challenging or impossible. With PAL_FINDER_v0.02.03, it is possible to partially avoid these loci. By only working with loci that qualify as pPALs, it is less likely the primers will amplify multiple loci. Even using the stringent criteria for pPALs, we found over 100 loci for each species, over 500 for 27 species, and over 1000 for 19 species. Overall, ~25% of all PALs qualify as pPALs.

**Figure 1:**
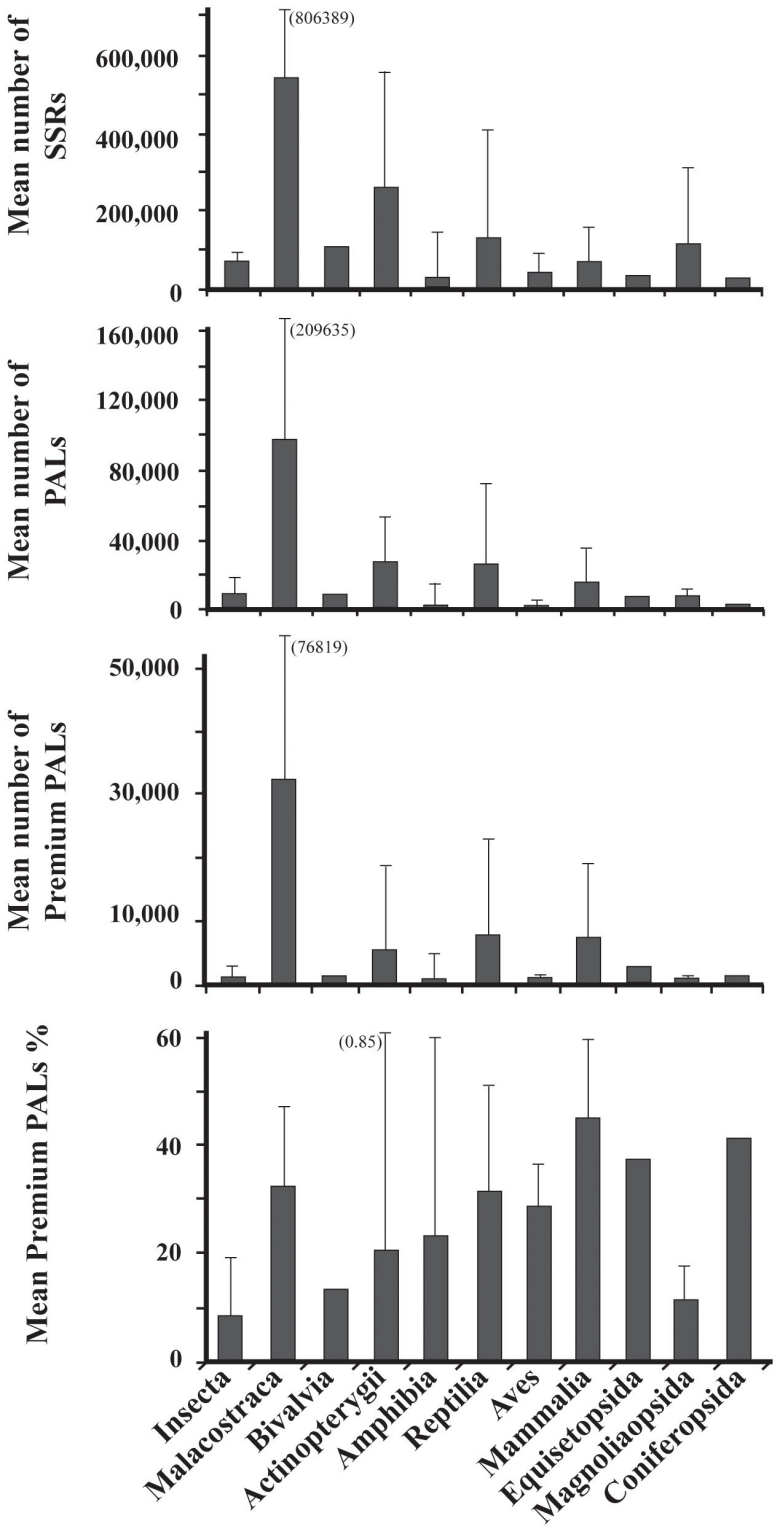
The mean and 95% upper confidence limit (values in parentheses are high values that go off the scale) for the number of SSR’s (a), PALs (b), pPALs (c), and percent of PALs that were pPALs that were observed across classes.

**Figure 2:**
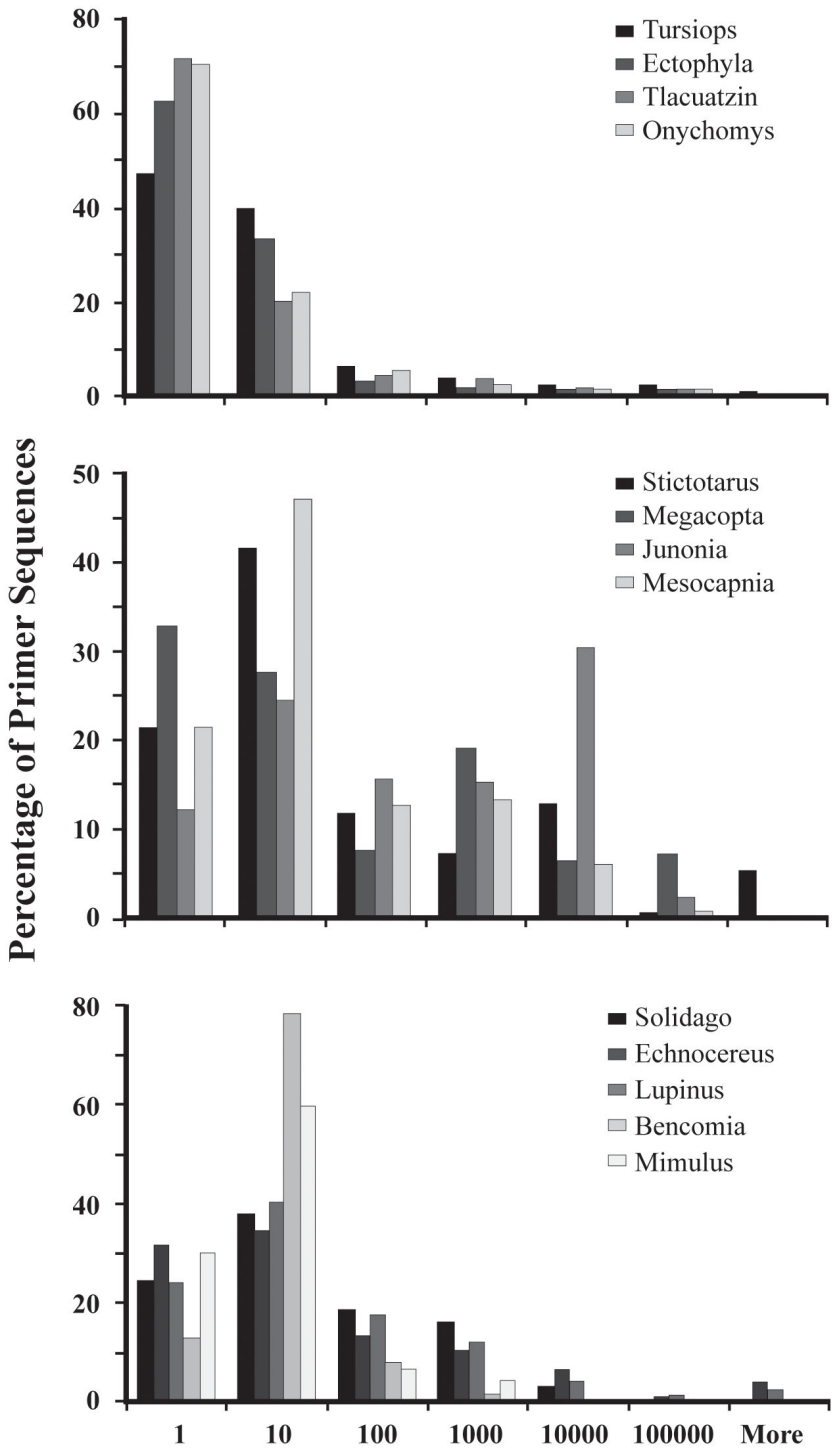
Frequency histograms of forward primer sequence copy number within 5 million paired end reads. The proportion of all primers observed 1, 2-10, 11-100, 101-1000, 1001-10,000, 10,001 – 100,000 or > 100,000 times is shown for Mammallia (a), Insecta (b), and Magnoliopsida (c).

Given the range of species included, we examined for effects of taxonomy on SSR development. There was no effect of kingdom or phylum on the number of SSRs, PALs, or pPALs found; however, class significantly affected all three categories (table 3). Across classes, the number of SSRs was lowest in the Amphibia and highest in Malacostraca. The number of PALs found was lowest in Aves and again highest in Malacostraca. However, for both measures there is ample variation across species within a class, as can be seen by the standard deviations (Figure 1a,b). The frequency of pPALs also ranged widely across taxa (mean = 5,607; range 136 - 52,682; table 4; Figure 1c). In working with PALs, the most important information is the proportion of PALs that are pPALs. Both phylum and class significantly affected this proportion (table 3), where the lowest proportion occurs in insects and the highest in mammals (Figure 1d). To further illustrate this point, we chose just one of the primer sequences (forward) and examined its copy number in the entire dataset. In some cases, the copy numbers of sequences is greater than 100,000 and frequently greater than 10,000 (Figure 2). In *Eurycea*, numerous primer sequences had copy numbers in excess of 900,000. Across taxa, the distribution of copy numbers is quite different. In 3 of 4 mammalian taxa tested, the copy number of most PALs is one and rarely exceeded 10 (Figure 2a). Contrast this with insects and plants within the class Magnoliopsida that have relatively high PAL copy numbers (Figure 2b and 2c). The benefit of using the IPE method in conjunction with PAL_FINDER v0.02.03 is the ability to identify and avoid these loci when desired. 

**Table 3:** Results of General Linear Model analysis examining role of taxonomy on the number of sequences that had microsatellites (No. msats), the number of PALs, the number of PALs that were different repeat types, the number of premium PALs (pPALs), the number of pPALs that were different repeat types, and the proportion of PALs that were pPALs.

	**Kingdom (2)**	**Phylum (7)**	**Class (11)**
**No. msats**	NS	NS	<0.0001
**No. PALs**	NS	NS	<0.0001
6mers	NS	NS	NS
5mers	NS	NS	NS
4mers	NS	NS	0.0491
3mers	NS	NS	0.0016
2mers	NS	0.05	<0.0001
**Premium PALS**	NS	NS	0.0003
6mers	NS	NS	NS
5mers	NS	NS	NS
4mers	0.06	NS	0.0061
3mers	NS	NS	0.0032
2mers	NS	NS	0.0001
**pPALs/PALs**	NS	0.0207	<0.0001

**Table 4:** Sample number and for each the number of pPALs found and the number that contained hexanucleotide, pentanucleotide, tetranucleotide, trinucleotide, or dinucleotide repeats.

**Sample Number**	**pPALs**	**6mers**	**5mers**	**4mers**	**3mers**	**2mers**
**1**	201	3	0	3	71	124
2	2,423	0	2	12	238	2,171
**3**	136	0	1	44	53	38
4	937	2	39	68	180	648
**5**	19,407	16	51	913	3,213	15,214
**6**	52,682	2	239	2,368	12,449	37,624
7	24,022	1	179	1,061	5,879	16,902
8	4,635	3	21	188	439	3,984
**9**	6,671	26	32	491	830	5,292
**10**	322	1	9	62	91	159
**11**	1,118	13	54	426	411	214
**12**	667	11	51	165	287	148
**13**	1,016	6	83	246	419	262
14	845	29	59	149	377	231
**15**	626	9	55	107	317	138
16	949	20	69	119	442	299
**17**	165	1	11	29	69	56
**18**	2,150	2	8	261	297	1,582
19	3,178	8	29	442	454	2,246
20	7,049	30	65	1,062	1,595	4,297
21	17,797	39	120	1,914	1,695	14,029
22	6,314	48	474	1,948	1,563	2,281
23	14,511	10	107	2,014	6,509	5,871
24	2,545	8	22	169	411	1,935
**25**	1,163	0	3	91	285	784
26	2,722	2	6	15	413	2,286
**27**	813	6	38	49	466	254
**28**	1,208	9	97	94	422	586
**29**	803	6	145	65	382	205
30	402	8	6	10	97	281
31	791	3	2	5	195	586
32	1,180	3	6	39	421	711

Interestingly, the types of SSRs found also varied across taxa. There was a significant effect of kingdom and phylum on the proportion of PALs and pPALs that were tetranucleotides, with fewer found in plants than animals (table 3). Class affected the proportion of most repeat types seen (table 3). As expected, dinucleotide repeats were overall the most common and accounted for > 50% of the SSRs for most species and classes (table 2). However when considering pPALs, Aves had relatively fewer dinucleotides and more hexa-, penta-, and tri-nucleotides than any other class. In amphibians, tetra-, tri-, and di-nucleotide repeats occurred at similar frequencies and had relatively more tetranucleotides than other classes. A vast majority of pPALs were dinucleotides in both fish species (83%) and the conifer (84%) species. However, due to the large number of SSRs identified, there are still numerous non-dinucleotide pPALs to work with (651 in *Rhinichthys*, 1379 in *Prosopium*, and 469 in *Juniperus*).

For the 13 species for which we optimized primers, we had clean amplification of a single locus for 61% of the loci when using a single set of pcr conditions and cycling parameters (table 5). Success varied across major groups with ~49%, 60%, and 67% amplifying in invertebrates, vertebrates, and plants respectively, with many other loci showing promise with additional optimization. One perceived problem with the IPE method is that once primers are designed the resulting amplicon size cannot be predicted. As we always designed primers in separate reads of the pair (i.e., forward primer in the forward read, and the reverse primer in the reverse read), and it was rarely the case that the paired ends overlapped, there was always uncertainty in how much sequence exists between the primers. Our methods only allowed us to visualize products under 550bp, thus it is possible that some primer pairs amplified larger fragments for which we could not detect. In some cases, the resulting product was too small for accurate sizing using our methods. This was a particular problem with the bivalve. However, we have ascertained that when the repetitive sequence was found in both of the paired reads the resulting amplicon is often very small, likely due to an overly short insert. After working with the bivalve, we began only ordering primers for loci in which the SSR was found in one direction only. This approach has eliminated short inserts, and subsequently short amplicons, as a serious problem. Alternatively, doing a strict size selection before sequencing could also remove these shorter loci. In general, for those species for which additional data on polymorphism and allelic diversity have been collected, a good spread of size ranges between 100 and 500bp have been observed [25-29]. The species that had the lowest success in yielding amplifiable loci was *Stictotarsus*. Interestingly, it also yielded a low proportion of pPALs, as well as very few tetranucleotide repeats, which in our experience amplify more cleanly. Developing robust SSR loci for Lepidopterans in general has been difficult, primarily due to the flanking sequences across loci being too similar ([30] and references therein). Often only a few loci are generated per species (e.g., [31-34]). In our own experience with earlier methods, we screened 96 primer pairs to obtain five loci [35]. In the current study, we screened 48 primer pairs for *Junonia coenia* using only a single set of amplification conditions and identified 26 loci that produced strong peaks and did not appear to amplify multiple loci. 

**Table 5:** Forty-eight primers were tested for amplification across 13 species.

**Amplification Result**	**Species Sample Number**												
	**1**	**2**	**3**	**4**	**5**	**8**	**9**	**10**	**11**	**21**	**24**	**25**	**31**
Number of loci with good amplification	11	24	26	25	19	23	29	11	22	29	40	11	30
Number of loci with good amplification, but were too small (e.g., <100bp)	0	3	2	0	0	1	5	6	3	4	1	24	1
Number of loci that would require further optimization	14	12	10	9	11	15	3	16	13	5	5	9	8
Number of loci that yielded zero amplification	23	9	10	14	18	9	11	15	10	10	2	4	8

Overall, our results demonstrate that Illumina paired-end sequencing identifies large numbers of SSR loci across a wide range of taxa. Additionally, using PAL_FINDER_v0.02.03 to analyze and refine the SSRs selection process, results in a high amplification success rate. In the current study we analyzed 5M reads per species, however, with sufficient resources much more data can be processed and we have now successfully analyzed up to 40M reads allowing for further refinement of PAL selection. 

Lastly, as both of our library preparation techniques yielded similar results, this IPE method is ideal even when only a very small amount of genomic DNA is available. 

## References

[1] Goldstein DB, Schlötterer C (1999) Microsatellites: Evolution and Applications. New York: Oxford University Press. 368 pp.

[2] Chistiakov DA, Hellemans B, Volckaert FAM (2006) Microsatellites and their genomic distribution, evolution, function and applications: A review with special reference to fish genetics. Aquaculture 255: 1-29. doi:10.1016/j.aquaculture.2005.11.031.

[3] Xing C, Schumacher FR, Xing G, Wang T, Elston RC (2005) Comparison of microsatellites, single nucleotide polymorphisms (SNPs) and composite markers derived from SNPs in linkage analysis. BMC Genet 6: S29. doi:10.1186/1471-2156-6-S1-S29. PubMed: 16451638. 16451638 PMC1866757

[4] Ogden R (2011) Unlocking the potential of genomic technologies for wildlife forensics. Mol Ecol Resour 11: 109–116. doi:10.1111/j.1755-0998.2010.02954.x. PubMed: 21429167. 21429167

[5] Baird NA, Etter PD, Atwood TS, Currey MC, Shiver AL et al. (2008) Rapid SNP Discovery and Genetic Mapping Using Sequenced RAD Markers. PLOS ONE 3(10): e3376. doi:10.1371/journal.pone.0003376. PubMed: 18852878. 18852878 PMC2557064

[6] Hohenlohe PA, Bassham S, Etter PD, Stiffler N, Johnson EA et al. (2010) Population Genomics of Parallel Adaptation in Threespine Stickleback using Sequenced RAD Tags. PLoS Genet 6(2): e1000862. doi:10.1371/journal.pgen.1000862. PubMed: 20195501. 20195501 PMC2829049

[7] Hohenlohe PA, Amish SJ, Catchen JM, Allendorf FW, Luikart G (2011) Next-generation RAD sequencing identifies thousands of SNPs for assessing hybridization between rainbow and westslope cutthroat trout. Mol Ecol Resour 11: 117–122. doi:10.1111/j.1755-0998.2010.02967.x. PubMed: 21429168. 21429168

[8] Santana QC, Coetzee MPA, Steenkamp ET, Mlonyeni OX, Hammond GNA et al. (2009) Microsatellite discovery by deep sequencing of enriched genomic libraries. BioTechniques 46: 217-223. doi:10.2144/000113085. PubMed: 19317665. 19317665

[9] Breton JS, Oliveira K, Drew RE, Jones KL, Hagen C et al. (2011) Development and characterization of ten polymorphic microsatellite loci in the yellowtail flounder (*Limanda* *ferruginea*). Conserv Genet Resour 3: 369-371. doi:10.1007/s12686-010-9364-5.

[10] Kwiatkowski MA, Somers CM, Poulin RG, Rudolph DC, Martino J et al. (2010) Development and characterization of 16 microsatellite markers for the Louisiana pine snake, *Pituophis* *ruthveni*, and two congeners of conservation concern. Conserv Genet Resour 2: 163-166. doi:10.1007/s12686-010-9208-3.

[11] Lance SL, Light JE, Jones KL, Hagen C, Hafner JC (2010) Isolation and characterization of 17 polymorphic microsatellite loci in the kangaroo mouse, genus *Microdipodops* (Rodentia: Heteromyidae). Conserv Genet Resour 2: 139-141. doi:10.1007/s12686-010-9195-4.

[12] Nunziata SO, Scott DE, Jones KL, Hagen C, Lance SL (2011) Twelve novel microsatellite markers for the marbled salamander, *Ambystoma* *opacum* . Conserv Genet Resour 3: 773-775. doi:10.1007/s12686-011-9455-y.

[13] Flanagan SP, Wilson WH, Jones KL, Lance SL (2010) Development and characterization of twelve polymorphic microsatellite loci in the Bog Copper, *Lycaena* *epixanthe* . Conserv Genet Resour 2: 159-161. doi:10.1007/s12686-010-9206-5.

[14] Henningsen JP, Lance SL, Jones KL, Hagen C, Laurila J et al. (2010) Development and characterization of 17 polymorphic loci in the faucet snail, *Bithynia* *tentaculata* (Gatropoda: Caenogastropoda: Bithyniidae). Conserv Genet Resour 2: 247-250. doi:10.1007/s12686-010-9255-9.

[15] Somers CM, Neudorf K, Jones KL, Lance SL (2011) Novel microsatellites for the compost earthworm *Eisenia* *fetida*: a genetic comparison of three North American vermiculture stocks. Pedobiologia 54: 111-117. doi:10.1016/j.pedobi.2010.11.002.

[16] Matesanz S, Sultan SE, Jones KL, Hagen C, Lance SL (2011) Development and characterization of microsatellite markers for *Polygonum* *cespitosum* (Polygonaceae). Am J Bot 98: e180-e182. doi:10.3732/ajb.1100053. PubMed: 21700804. 21700804

[17] Allen JM, Obae SG, Brand MH, Silander JA, Jones KL et al. (2012) Development and characterization of microsatellite markers for *Berberis* *thunbergii* (Berberidaceae). Am J Bot 99(5): e220-e222. doi:10.3732/ajb.1100530. PubMed: 22542902. 22542902

[18] Tay WT, Behere GT, Batterham P, Heckel DG (2010) Generation of microsatellite repeat families by RTE retrotransposons in lepidopteran genomes. BMC Evol Biol 10: 144. doi:10.1186/1471-2148-10-144. PubMed: 20470440. 20470440 PMC2887409

[19] Abdelkrim J, Robertson B, Stanton JA, Gemmell N (2009) Fast, cost-effective development of species-specific microsatellite markers by genomic sequencing. BioTechniques 46: 185-192. doi:10.2144/000113084. PubMed: 19317661. 19317661

[20] Castoe TA, Poole AW, Gu W, de Koning APJ, Daza JM et al. (2010) Rapid identification of thousands of copperhead snake (*Agkistrodon* *contortrix*) microsatellite loci from modest amounts of 454 shotgun genome sequence. Mol Ecol Resour 10: 341-347. doi:10.1111/j.1755-0998.2009.02750.x. PubMed: 21565030. 21565030 PMC5172459

[21] Castoe TA, Poole AW, de Koning APJ, Jones KL, Tomback DF et al. (2012) Rapid microsatellite identification from Illumina paired-end genomic sequencing in two birds and a snake. PLOS ONE 7(2): e30953. doi:10.1371/journal.pone.0030953. PubMed: 22348032. 22348032 PMC3279355

[22] Warren WC, Clayton DF, Ellegren H, Arnold AP, Hillier LW et al. (2010) The genome of a songbird. Nature 432: 695-716. PubMed: 20360741.

[23] Don RH, Cox PT, Wainwright BJ, Baker K, Mattick JS (1991) ‘Touchdown’ PCR to circumvent spurious priming during gene amplification. Nucleic Acids Res 19: 4008. doi:10.1093/nar/19.14.4008. PubMed: 1861999. 1861999 PMC328507

[24] DeWoody AJ, Schupp J, Kenefic L, Busch J, Murfitt L et al. (2004) Universal method for producing ROX-labeled size standards suitable for automated genotyping. BioTechniques 37: 348-350. PubMed: 15470886. 15470886 10.2144/04373BM02

[25] Nunziata SO, Karron JD, Mitchell RJ, Lance SL, Jones KL et al. (2012) Characterization of 42 polymorphic nuclear microsatellite loci in *Mimulus* *ringens* (Phrymaceae) using Illumina sequencing. Am J Bot 12: e477-e480. 10.3732/ajb.1200180 23196400

[26] Nunziata SO, Lance SL, Jones KL, Nerkowski S, Metcalf AE (2013) Development and characterization of twenty-three microsatellite markers for the freshwater minnow Santa Ana Speckled Dace (Rhinichthys osculus spp., Cyprinidae) using paired-end Illumina shotgun sequencing. Conserv Genet Resour 5: 145-148. doi:10.1007/s12686-012-9754-y.

[27] O’Bryhim J, Chong JP, Lance SL, Jones KL, Roe KJ (2012) Development and characterization of sixteen microsatellite markers for the federally endangered species: *Leptodea* *leptodon* (Bivalvia: Unionidae) using paired-end Illumina shotgun sequencing. Conserv Genet Resour 4(3): 787-789. doi:10.1007/s12686-012-9644-3.

[28] O’Bryhim J, Somers C, Lance SL, Yau M, Boreham DR, Jones KL et al. (2013) Development and characterization of twenty-two novel microsatellite markers for the mountain whitefish, *Prosopium* *williamsoni* and cross-amplification in the round whitefish, *P*. *cylindraceum*, using paired-end Illumina shotgun sequencing. Conserv Genet Resour 5: 89-91. doi:10.1007/s12686-012-9740-4.

[29] Stoutamore JL, Love CN, Lance SL, Jones KL, Tallmon D (2012) Development of polymorphic microsatellite markers for the blue king crab (*Paralithodes* *platypus*). Conserv Genet Resour 4: 897-899. doi:10.1007/s12686-012-9668-8.

[30] Van’t Hof AE, Brakefield PM, Saccheri IJ, Zwaan BJ (2007) Evolutionary dynamics of multilocus microsatellite arrangements in the genome of the butterfly *Bicyclus* *anynana*, with implications for other Lepidoptera. Heredity (Edinb) 98: 320-328. doi:10.1038/sj.hdy.6800944. PubMed: 17327875. 17327875

[31] Meglécz E, Solignac M (1998) Microsatellite loci for *Parnassius* *mnemosyne* (Lepidoptera). Hereditas 128: 179-180.

[32] Keyghobadi N, Roland J, Strobeck C (1999) Influence of landscape on the population structure of the alpine butterfly *Parnassius* *smintheus* (Papilionidae). Mol Ecol 8: 1482-1495. 10.1046/j.1365-294x.1999.00726.x 10564454

[33] Reddy KD, Abraham EG, Nagaraju J (1999) Microsatellites in the silkworm, *Bombyx* *mori*: abundance, polymorphism, and strain characterization. Genome 42: 1057-1065. doi:10.1139/gen-42-6-1057. PubMed: 10659770. 10659770

[34] Harper GL, Piyapattanakorn S, Goulson D, Maclean N (2000) Isolation of microsatellite markers from the Adonis blue butterfly (*Lysandra* *bellargus*). Mol Ecol 9: 1948-1949. doi:10.1046/j.1365-294x.2000.01097-17.x. PubMed: 11091345. 11091345

[35] Milko LV, Haddad NM, Lance SL (2012) Dispersal via stream corridors structures populations of the endangered St. Francis’ satyr butterfly (*Neonympha* *mitchelli* *francisci*). J Insect Conserv 16: 263-273. doi:10.1007/s10841-011-9413-8.

